# High‐resolution HLA genotyping identifies alleles associated with severe COVID‐19: A preliminary study from India

**DOI:** 10.1002/iid3.481

**Published:** 2021-07-21

**Authors:** Ravikanth Vishnubhotla, Mitnala Sasikala, Vijayasarathy Ketavarapu, Duvvur Nageshwar Reddy

**Affiliations:** ^1^ Asian Healthcare Foundation/AIG Hospitals Hyderabad Telangana India

**Keywords:** COVID‐19, HLA genotyping, SARS‐CoV‐2, severe COVID‐19, vaccination

## Abstract

**Introduction:**

Human leukocyte antigen (HLA) variability has been demonstrated to be associated with susceptibility/severity of COVID‐19. High‐resolution HLA genotyping to identify alleles associated with severe COVID‐19 in an Indian cohort was performed.

**Methods:**

Quantitative reverse‐transcription polymerase chain reaction‐confirmed SARS‐CoV‐2‐positive patients with mild/moderate/severe disease (*n* = 54) and asymptomatic (*n* = 42) were recruited and genotyped for 11‐HLA loci on MiSeq using NGSgo®‐MX11‐3 and analyzed (NGSengine; GenDx).

**Results:**

A significant difference in alleles between the groups was identified for HLA‐C*04:01:01:01, HLA‐DRB5*01:01:01:02, HLA‐DQA1*03:01:01:01, HLA‐DPB1*04:01:01:41, and HLA‐DPA1*01:03:01:02. Alleles namely, HLA‐C*04:01:01:01 (OR: 5.71; 95% CI: 1.2–27.14; *p* = .02), HLA‐DRB5*01:01:01:02 (OR: 2.94; 95% CI: 1.1–7.84; *p* = .03), DQA1*03:01:01:01 (OR: 22.47; 95% CI: 1.28–393.5; *p* = .03), HLA‐DPB1*04:01:01:41 (OR: 9.44; 95% CI: 0.5–175.81; *p* = .13), and HLA‐DPA1*01:03:01:02 (OR: 8.27; 95% CI: 2.26–30.21; *p* = .001) were associated with severe COVID‐19.

**Conclusion:**

Genotyping for these alleles will enable identification of individuals at risk of severe disease and stratification for preferential vaccination.

## 1 INTRODUCTION

The current COVID‐19 pandemic is caused by the newly emerged positive‐stranded RNA virus SARS‐CoV‐2.^1^ The virus enters host cells by fusion of membranes, a crucial step in establishing infection leading to acute respiratory distress syndrome.^2^ The severity of the disease depends on the virulence of the strain, genetic determinants, host entry factors/immunity and pre‐existing comorbidities. The importance of the host immune system in viral clearance is well documented, and variability in human leukocyte antigens (HLAs), which drive immune responses, have been shown to be associated with the severity of COVID‐19.^3^ Most of these studies have been based on bioinformatics approaches. Such predictions have limited clinical implications and ascertain the need for high‐resolution HLA typing in COVID‐19 patients. On the basis of these observations, we aimed to identify HLA alleles that are associated with a risk of severe COVID‐19 disease.

## 2 MATERIALS AND METHODS

This was a single‐center observational study conducted with approval from the Institutional Ethics Committee of AIG Hospitals, Hyderabad. Subjects who attended the COVID‐19 testing center from August 30, 2020 to September 7, 2020 were screened for SARS‐CoV‐2 by quantitative reverse‐transcription polymerase chain reaction. Patients whose nasopharyngeal swabs were positive (*n* = 48) and who did not have any symptoms for 15 days or more were classified as asymptomatic. Patients who developed symptoms were classified in to mild/moderate or severe based on severity according to the diagnostic criteria of the Diagnostic and Therapeutic Program of Novel Coronavirus Pneumonia (Sixth version for trial implementation).^4^ All the participants provided informed consent. Whole blood (5 ml) was collected for standard investigations and DNA isolation for HLA typing. DNA was isolated from the samples using the HiPurA™ SPP Blood DNA Isolation Kit (Himedia), following the manufacturer's protocol. The quality and quantity of DNA were assessed using a Nanodrop spectrophotometer (Thermo Fisher Scientific™). High‐resolution HLA genotyping was performed on a next‐generation sequencer (Illumina MiSeq) employing the NGSgo®‐MX11‐3 kit (GenDx), following the manufacturer's protocol. Briefly, the protocol involved locus‐specific amplification of the complete HLA region in three pools: Pool 1 targeting the regions HLA‐A (3.1 kb), HLA‐DRB1 (7.5 kb), HLA‐DPB1 (5 kb), HLA‐DRB3 (7.5 kb), HLA‐DQA1 (5.8 kb); pool 2 targeting HLA‐B (3.4 kb), HLA‐DQB1 (6.7 kb), HLA‐DRB5 (7.4 kb), and pool 3 targeting HLA‐C (3.4 kb), HLA‐DPB1 (5.7 kb), HLA‐DRB4 (4.3 kb), and HLA‐DPB1 (5.5 kb). Approximately 60 ng of the isolated DNA was amplified in three pools. Generated sequences were assessed for quality, sequenced and the alleles were interpreted using NGSengine; GenDx. Multiple Logistic regression analysis was applied to the variables that were significant in univariate analysis to estimate the risk of susceptibility to COVID‐19. T‐cell epitope prediction for the binding of the identified HLA alleles on the spike protein of the SARS‐CoV‐2 was carried out using Immune Epitope Database and Analysis Resource (https://www.iedb.org/home_v3.php). The protein sequence of the spike of SARS‐CoV‐2 was downloaded from https://www.uniprot.org/uniprot/P0DTC2.fasta. Allele frequencies for the significant HLA alleles were retrieved from http://allelefrequencies.net/.

## 3 RESULTS AND DISCUSSION

The clinical characteristics of the asymptomatic and symptomatic (mild to moderate/severe) COVID‐19 patients are presented in Table 1. Association analysis between the two groups identified a total of five alleles to be significantly different between the groups. HLA‐C*04:01:01:01, HLA‐DRB5*01:01:01:02, HLA‐DQA1*03:01:01:01, HLA‐DQA1*01:03:01:02, and HLA‐DPB1*04:01:01:41 conferred an enhanced risk of mild to moderate/severe disease (Table 2). Multivariate analysis confirmed the risk of severe COVID‐19 disease associated with the identified alleles and the data is as presented in Table 3. T‐cell epitope prediction for the spike protein of the SARS‐CoV‐2 identified that the risk conferring alleles had the fewest predicted binding peptides.

**Table 1:** Clinical characteristics of the study group

Parameter	Asymptomatic (*n* = 42)	Symptomatic (*n* = 54)	Significance
Age (years) (mean ± *SD*)	32.83 ± 10.26	52.65 ± 13.96	.0001
Age range (years)	16–57	26–85	‐
Gender (male), *n* (%)	26 (61.90)	40 (74.07)	.02
Comorbidities, *n* (%)			
Normal	42 (100)	21 (38.89)	.00001
Only diabetes	0	10 (18.52)	.003
Only hypertension	0	4 (7.41)	.07
Diabetes/hypertension	0	19 (35.19)	.00001
Blood counts			
Haemoglobin (g/dl)	13.74 ± 2.14	15.81 ± 17.28	.44
RBC (cells/mcl)	5.03 ± 0.63	4.62 ± 0.61	.001
WBC cells/µl	8376.92 ± 2941.93	8670.59 ± 5039.45	.73
Neutrophils (%)	56.08 ± 12.07	71.79 ± 12.54	.0001
Lymphocytes (%)	35.56 ± 10.58	20.50 ± 11.41	.91
Eosinophils (%)	2.90 ± 1.35	1.59 ± 1.31	.0001
Monocytes (%)	5.36 ± 2.64	5.59 ± 2.26	.64
Platelets (mcl)	2.99 ± 0.81	2.34 ± 0.70	.0001
Oxygen delivery			
On room air, *n* (%)	None	30 (55.56)	‐
On face mask, *n* (%)	None	2 (3.70)	‐
Nasal prongs, *n* (%)	None	4 (7.40)	‐
On noninvasive ventilation, *n* (%)	None	6 (11.12)	‐
On high‐flow nasal cannula, *n* (%)	None	1 (1.85)	‐
Non‐rebreather mask, *n* (%)	None	2 (3.70)	‐
Ventilator, *n* (%)	None	9 (16.67)	‐
FiO_2_	Not done	43.44 ± 26.64	‐
Serum ferritin level (ng/ml)	Not done	554.90 ± 533.92	‐
Interleukin‐6 levels (pg/ml)	Not done	108.56 ± 151.52	‐
D‐dimer (ng/ml)	<200	1024.28 ± 1385.8	‐
Chest X‐ray, *n* (%)			
Normal	33 (78.57)	2 (3.71)	.00001
Unilateral infiltration	9 (21.43)	14 (25.92)	.61
Bilateral infiltration	0 (0)	38 (70.37)	.00001

Abbreviation: g/dL, grams per decilitre; cells/mcL, cells per microliter; μl, microliter; %, percentage; FiO2, fraction of inspired oxygen; ng, nanogram; ml, milli litre; pg, picogram.

**Table 2:** Association of human leukocyte antigen alleles with severity

		Asymptomatic		Symptomatic				95% CI		
Allele	Genotype	Absent	Present	Absent	Present	*p* Value*	Odds ratio	Lower	Upper	*p* Value
HLA‐C	04:01:01:01	40	2	42	12	.01	5.71	1.2	27.14	.02
DRB5	01:01:01:02	35	7	34	20	.02	2.94	1.1	7.84	.03
DQA1	03:01:01:01	42	0	43	11	.001	22.47	1.28	393.5	.03
DPB1	04:01:01:41	42	0	49	5	.04	9.44	0.5	175.81	.13
DPA1	01:03:01:02	39	3	33	21	.0004	8.27	2.26	30.21	.001

* Chi‐square test was used.

**Table 3:** Results of multiple logistic regression analysis

	Regression coefficient	Standard error	Odds ratio		95% CI	
				*p* Value	Lower	Upper
Age	2.04	0.57	7.73	.004	2.51	23.77
Gender	0.47	0.58	1.60	.41	0.51	5.02
DPA1*01:03:01:02	2.22	0.90	9.21	.01	1.55	54.81
DPB1*04:01:04:41	−0.95	1.10	2.59	.38	0.29	22.59
DRB5*01:01:01:02	1.47	0.62	4.39	.01	1.27	15.06
HLA‐C*04:01:01:01	2.39	1.05	11.01	.02	1.38	87.43
Constant	−1.39			.0001		

High‐resolution HLA typing and comparison of alleles between asymptomatic and symptomatic patients may provide insights into efficient antigen presentation and viral clearance. Although it is known that HLA alleles influence disease susceptibility and/or severity of COVID‐19, the association of specific alleles with symptomatic patients is not documented from the Indian ethnicity. In this study, we performed high‐resolution HLA genotyping to identify the predominant alleles associated with the risk of mild to moderate/severe COVID‐19 disease.

Global distribution of the frequency of the HLA‐C*04:01:01:01 allele suggests that it is predominantly seen in Central America (as high as 0.52 to as low as 0.02) followed by the North American population (0.10–0.16). It is reported at a frequency of 0.21 in the Indian Tamil Nadar population, however, it is reportedly low in the other Indian ethnicities which is confirmed by the current study. It is reportedly low in the South‐East and Western Asian ethnicity (0.07) and completely absent in the North African ethnicity. We report the risk associated with HLA‐C*04:01:01:01 and development of symptoms in COVID‐19 patients and also confirm earlier findings from a multi‐ethnic group (Germany, Spain, Switzerland, and the United States) that identified an enhanced risk of requirement for intubation in COVID‐19 patients.^5^

Global distribution frequency for the other alleles at the 4‐field level of resolution could not be retrieved. DRB5*01:01:01:02 is a recently identified allele in a Chinese individual and differs from DRB5*01:01:01 by changes in the intron 2 at four positions.^6^ An earlier study identified the association of HLA‐DQA1*03:01 with enhanced risk of Celiac disease and Type‐1 diabetes. The increased risk was hypothesized to be due to the putative presence of DQ heterodimers that are encoded by the alleles in trans, apart from the DQ molecules encoded by the alleles in *cis*, on the cell surface of the immune cells.^7^ HLA‐DQA1*01:03 is associated with Bullous pemphigoid an autoimmune disease in Brazilian population.^8^

Multiple different loci are reported in various ethnicities that confer enhanced susceptibility to severe disease in COVID‐19. A recent study carried out in the Saudi Arabian population reported an increased frequency of HLA‐A*01, B*56, and C*01 among infected patients as compared to the controls. In addition, the study also reported an increased frequency of HLA‐A*03 and C*06 among patients with fatal outcomes as compared to infected patients. HLA‐DRB1*04 was also reportedly higher in the control group compared to infected patients conferring protection, while HLA‐DRB1*08 was associated with conferring susceptibility.^9^ Another study that performed high‐resolution sequencing for eight HLA genes in the Japanese ethnicity identified the association of HLA‐A*11:01:01:01 and HLA‐C*12:02:02:01–HLA‐B*52:01:01:02 with the severity of COVID‐19.^10^ HLA typing in Italian patients identified the association of HLA‐DRB1*15:01, ‐DQB1*06:02, and ‐B*27:07 with susceptibility to COVID‐19.^11^ Although there is variability in the alleles conferring susceptibility to COVID‐19 across ethnicities, the inability of the identified alleles to effectively bind the SARS‐CoV‐2 viral peptides among others may be one of the main reasons for severe disease.

Our pilot study involving a small sample size demonstrates the association of HLA alleles with mild to moderate/severe COVID‐19 disease. Furthermore, T‐cell epitope prediction identified that the risk conferring alleles had the fewest predicted binding peptides for the spike protein of the SARS‐CoV‐2, to present the highly conserved SARS‐CoV‐2 viral peptides. This reiterates that the identified alleles might have a role in conferring risk of severe disease and, therefore, clinically important. These results need to be validated in multiethnic larger sample so that they can be employed for routine genotyping as part of risk and severity assessment in future.

## CONFLICT OF INTERESTS

The authors declare that there are no conflict of interests.

## Data Availability

The data that support the findings of this study are available from the corresponding author upon reasonable request.
